# A Live Video Resiliency Dyadic Intervention for Persons With Dementia and Their Care-Partners Early After Diagnosis: Protocol for Open Pilot of Resilient Together for Dementia

**DOI:** 10.2196/60382

**Published:** 2025-01-15

**Authors:** Sydney McCage, Kristin Walker, Talea Cornelius, Robert A Parker, Kristen Dams-O'Connor, Brad Dickerson, Christine Ritchie, Ana-Maria Vranceanu, Sarah Bannon

**Affiliations:** 1 Brain Injury Research Center Department of Rehabilitation and Human Performance Icahn School of Medicine at Mount Sinai New York, NY United States; 2 Department of Psychology Stony Brook University Stony Brook, NY United States; 3 Department of Medicine Columbia University Irving Medical Center New York, NY United States; 4 Biostatistics Center Massachusetts General Hospital Department of Medicine Harvard Medical School Boston, MA United States; 5 Department of Neurology Icahn School of Medicine at Mount Sinai New York, NY United States; 6 Frontotemporal Disorders Unit Departments of Neurology and Psychiatry Massachusetts General Hospital and Harvard Medical School Boston, MA United States; 7 Department of Medicine Mongan Institute Center for Aging and Serious Illness and the Division of Palliative Care and Geriatric Medicine Massachusetts General Hospital and Harvard Medical School Boston, MA United States; 8 Center for Health Outcomes and Interdisciplinary Research Department of Psychiatry Massachusetts General Hospital and Harvard Medical School Boston, MA United States; 9 Department of Geriatrics and Palliative Medicine Icahn School of Medicine at Mount Sinai New York, NY United States

**Keywords:** dementia, dyad, emotional distress, intervention, diagnosis, telehealth, resilient, dyadic intervention, care-partner, Alzheimer’s disease, ADRD, psychosocial, depression

## Abstract

**Background:**

Alzheimer disease and related dementias (ADRDs) are increasingly common progressive conditions that have a substantial impact on individuals and their primary care partners—together described as a *dyad*. The stressors experienced by dyad members at around the time of ADRD diagnosis commonly produce clinically elevated emotional distress (ie, depression and anxiety symptoms), which can become chronic and negatively impact health, relationships, and the overall quality of life. Dyads commonly report unmet needs for early support to address these challenges early after diagnosis.

**Objective:**

This study is part of a larger study that has the primary objective to develop, adapt, and establish the feasibility of Resilient Together for Dementia (RT-ADRD), a novel dyadic skills-based intervention aimed at preventing chronic emotional distress early after diagnosis. The present study protocol describes an open pilot of the RT-ADRD intervention. This study will allow the study team to gather feedback on intervention components, administration of study measures, issues within general protocol, and perceptions about live video interventions prior to a larger feasibility trial.

**Methods:**

All study procedures will be conducted on the web (via phone and health care system–supported videoconferencing) to optimize accessibility, inclusion, and representativeness. Eligible dyads will include couples (up to N=10) referred from Mount Sinai Hospital (MSH) clinics within 3 months of an ADRD diagnosis. Dyads will be referred by their diagnosing clinicians (eg, neurologists, geriatricians, and neuropsychologists) and screened for eligibility. Eligible dyads will have at least one member who exhibits clinically elevated emotional distress and will demonstrate capacity to consent to research participation on a standardized assessment. Consenting dyads will complete baseline assessments of emotional distress, quality of life, relationship functioning, and resiliency skills. Dyads will then participate in 6 weekly RT-ADRD sessions together (30-60 minutes each). After the conclusion of the intervention, dyad members will complete posttest assessments with similar measures as the pretest. Finally, dyads will participate together in a single 60-minute exit interview to gather information on intervention content and procedures to refine the intervention before a pilot feasibility trial.

**Results:**

This study has been approved by the MSH institutional review board and is registered on ClinicalTrials.gov (NCT06421545). We anticipate that the study will be completed by late 2024.

**Conclusions:**

We will use these results to administer changes and develop procedures for a pilot feasibility trial of RT-ADRD relative to a minimally enhanced control condition. Our study will allow us to gather comprehensive information on proposed RT-ADRD procedures and content and the best ways of delivering prevention-focused interventions to reduce the potential for chronic emotional distress stemming from ADRDs.

**International Registered Report Identifier (IRRID):**

DERR1-10.2196/60382

## Introduction

### Background

Alzheimer disease and Alzheimer disease-related dementias (ADRDs) are conditions characterized by progressive declines in memory, language, behavior, and personality and produce early symptoms that include increased forgetfulness, communication challenges, and loss of recognition of places, time, and routines [1-3]. These early symptoms tend to worsen over time and undermine the independence, health, and well-being of persons living with ADRDs and their family care partners, many of whom are romantic or spousal partners [4-6]. Because both individuals and their partners (often referred to as “the second patient”) experience life disruptions stemming from ADRD symptoms that amplify stress, role changes, and relationship strain [6,7], both individuals and their romantic partners require early support [8]. This need is ever more pressing; by 2030, it is expected that 75 million individuals will be living with ADRD worldwide [5].

In the time following the onset of ADRD symptoms and particularly after receiving a formal diagnosis, many individuals (23%-53%) and their care partners (35%-50%) experience clinically elevated emotional distress (ie, depression and anxiety symptoms) [9-13]. These elevated rates are not surprising given the poor prognosis and lack of disease-modifying interventions for ADRDs, limited resources to assist with early challenges, difficulties engaging in social support, and changes to established roles and responsibilities (eg, caretaking) [14-16]. Early emotional distress is an important intervention target in dyads facing ADRDs for several reasons. First, distress is *interdependent* (ie, correlated and bidirectionally influenced) within dyads. Second, untreated distress is likely to become chronic, and chronic emotional distress is difficult to treat and linked to the deterioration of dyads’ physical and mental health, relationship quality, and quality of life [9,14,17,18]. Third, distress interferes with dyads’ ability to effectively communicate and cope with early challenges linked to ADRDs, which can impact long-term adjustment [9,14].

Early psychosocial interventions may help individuals with ADRDs and their care partners adjust to challenges and plan collaboratively for the future, which can alleviate distress and maintain overall relationship quality and well-being [8]. Existing interventions for early or moderate-stage ADRDs primarily target family care partners, and several interventions demonstrate lasting improvements in depression symptoms and quality of life [5]. Dyads commonly express strong preferences to participate in interventions together, and dyadic interventions in the first few years of ADRD diagnosis demonstrate promise in reducing dyads’ emotional distress as well as neuropsychiatric symptoms expressed by the person living with ADRD [19]. Interventions have historically ignored the “window of opportunity” to meaningfully include persons with ADRDs in interventions shortly after diagnosis, limiting their potential to assist in collaborative treatment and planning before symptoms progress [20-22]. Currently, there are no dyadic psychosocial interventions that prioritize delivery in the first weeks and months after diagnosis to promote dyadic adjustment to ADRDs [20,23-25].

**Figure 1:**
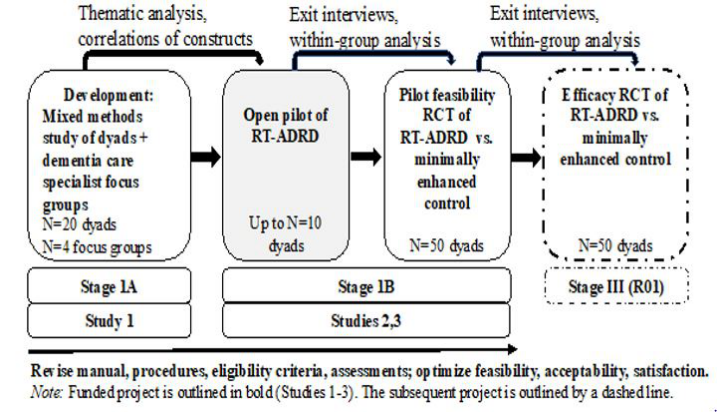
Iterative Development of RT-ADRD (Resilient Together for Dementia).

Dyadic interventions may be a more effective, efficient, and economical avenue for promoting positive adjustment to ADRDs for individuals and their care partners earlier in the course of the illness [24,26]. Our team has developed successful dyadic interventions to prevent distress following medical adversity, including Recovering Together, a 6-session dyadic resiliency intervention that addresses emotional distress in patients and informal care partners shortly after neurointensive care unit admission for acute neurological conditions [27,28]. Recovering Together program uses interpersonal theories of adjustment aimed toward preventing chronic stress and transdiagnostic resiliency skills that we expect could also serve as mechanistic treatment targets for other neurological conditions such as ADRDs [29-37]. We then leveraged the NIH (National Institutes of Health) Stage Model for intervention development and in-depth interviews with ADRD clinician experts and dyads (Figure 1) to inform the development of a similar intervention to address early challenges linked to ADRDs [38,39].

### This Study

This study protocol is part of a larger 5-year study that aims to use the prior research, methodology, program content, and procedures of the Recovering Together program as a basis for developing the novel Resilient Together for Dementia (RT-ADRD). Our primary objective is to describe the protocol for the open pilot study of RT-ADRD that will inform a subsequent pilot feasibility trial of the program relative to a control condition (NIH Stage Model Stage 1a). In this study, we aim to gather comprehensive information from dyads regarding the feasibility of assessment, feedback on the intervention components, applicability of study measures, and to identify potential issues prior to the feasibility trial.

## Methods

### Ethical Considerations

This open pilot study was approved by the Icahn School of Medicine at Mount Sinai Independent Review Board (23-01360) and is registered on ClinicalTrials.gov (NCT06421545). Participants will provide electronic consent to participate via the REDCap (Research Electronic Data Capture; Vanderbilt University) HIPAA (Health Insurance Portability and Accountability Act)-compliant web-based application using a hospital-approved e-consent feature. Prospective participants will be given ample time to review the contents of the consent form as well as speak with the study coordinator who will provide detailed information about the study and answer any questions. The participants will also be reminded that they may decline to participate and can discontinue participation at any time. Participants will be given a copy of the consent form and will be invited to ask questions about their participation at any point over the course of the study. The identity of the participants will not be revealed in the presentation or publication of any results from this project. To protect confidentiality, all data will be deidentified and only identifiable by subject codes. Participants will be individually compensated US $80 for completion of the pre- and posttest assessments, for a total of US $160 per dyad at the end of the program.

### Study Design

We will conduct an open pilot of RT-ADRD dyadic intervention involving dyads comprising (1) individuals with a recent ADRD diagnosis and (2) their spousal or romantic care partners, with a target recruitment of up to N=10 dyads, or 20 total participants. This study is designed to gather data that can be used to revise the intervention before a larger pilot feasibility trial.

### Inclusion and Exclusion Criteria

Eligible participants will be adult dyads who are partners that currently live together and are willing to participate in the RT-ADRD intervention that are (1) English speaking, (2) patient with newly diagnosed (within 3 months) with an ADRD diagnosis after age 65 years (determined by medical chart review and provider referral), (3) patient with cognitive assessment scores and symptoms consistent with early-stage ADRD, as determined by Clinical Dementia Rating scale scores of 0.5 (very mild) or 1.0 (mild dementia) and Montreal Cognitive Assessment (MoCA) scores of 17-25, obtained via a review of medical records (if patients do not have cognitive assessment scores available, we will administer the MoCA to confirm eligibility), (4) either partner demonstrates clinically elevated emotional distress, as determined by Hospital Anxiety and Depression Scale [40], subscale scores >8 or Geriatric Depression Scale [41], scores > 5, and (5) both partners demonstrate the ability to understand study and research protocol, as determined by the University of California Brief Assessment of Capacity to Consent [42], >12. Individuals will not be eligible for the study if (1) either partner has a co-occurring terminal illness, and (2) patient was diagnosed with forms of dementia with clinical profiles that would preclude participation (eg, highly symptomatic Frontotemporal Dementia–behavioral variant), as determined by the treatment team.

### Study Procedures

Dyads will be recruited from the Mount Sinai Departments of Neurology, Geriatrics and Palliative Medicine, and the hospital system’s broader dementia clinical care infrastructure. We will circulate information via flyers, presentations, and word-of-mouth referrals, and dyads can be self-referred or be referred by their treating dementia care providers. The study team will present the aims to all staff at the clinics to explain the purpose of the study, potential benefits to participants, and discuss best ways to facilitate referrals. The study team obtained a waiver of informed consent for the purpose of eligibility screening and will also review electronic medical records of patients treated in relevant clinics to inform providers of potential eligibility prior to diagnostic disclosure visits. All recruitment, screening, and consent procedures will be performed remotely via telephone, electronic survey through REDCap, email, and over a live video using health care system–supported videoconferencing.

Treating providers will obtain permission from the dyad to be contacted by the study team. If self-referred, interested dyads will also have the option to respond to a brief survey via QR code or URL to a secure REDCap platform listed on the distributed study flyers. The study team will contact interested participants via telephone to schedule a telephone or live video call with the study research assistant, who will describe the study to potential participants and conduct screening for eligibility (including capacity to consent). If either dyad member is deemed ineligible based on screening criteria, the study team will provide dyad with a comprehensive list of resources containing local and national resources and document ineligibility in REDCap. If eligible and interested in participating, individuals will complete consent procedures by indicating verbal consent and documenting electronic consent a secure password-protected REDCap platform.

After enrollment, participants will be sent a baseline survey via a secure REDCap link to assess demographics, emotional distress, resiliency factors, and relationship functioning. Participants can also complete assessments via health care system–supported videoconferencing with the help of a trained research assistant. Once the dyad has completed the baseline survey, a member of the research team will schedule the first of 6 RT-ADRD treatment sessions together led by a PhD-level clinical psychologist with expertise in conducting dyadic interventions (SB). In advance of the treatment session, the dyad will be sent copy of the treatment manual to review the skills that will be taught during the 6-week treatment program. Each session will be audio recorded for the purpose of manual refinement. After completing treatment, dyad members will complete a similar set of surveys at posttest. Finally, they will participate in a single 60-minute exit interview together to gather comprehensive feedback on the material to further refine the intervention. Each dyad member will be compensated US $80 for completion of the pre- and posttest assessments, for a total of US $160 per dyad at the end of the program.

### RT-ADRD Intervention Content

The RT-ADRD intervention will have 6 weekly live video treatment sessions lasting 30-60 minutes each delivered over health care system–supported videoconferencing; in-person and hybrid session options will be available as well, according to dyad preference. The content and procedures were developed by adapting the Recovering Together dyadic resiliency intervention for neurointensive care unit admitted dyads [27], and then revised using feedback from interviews with dementia care specialists [38], and from dyads interviewed after ADRD diagnosis [39]. Sessions for RT-ADRD contain content and training in resiliency skills that are particularly relevant after ADRD diagnosis for patients and their care partners. RT-ADRD was designed to include individual and interpersonal coping skills that are *transdiagnostic* (ie, relevant across mental and physical health conditions) and drawn from prominent evidence-based therapy approaches (eg, mindfulness, Dialectical Behavior Therapy, and Cognitive Behavioral Therapy). The sessions emphasize ways of using skills to cope with the common challenges experienced early after dementia diagnosis and focus on navigating challenges as a dyadic unit Dyads will also receive links with downloadable recordings of exercises for home practice of skills. The sessions are modular in nature, and dyads will choose 6 modules from a selection of 8 possible modules. The 8 modules are presented in Table 1.

**Table 1:** Sessions’ outlines for the Resilient Together for Dementia (RT-ADRD) program.

Sessions	RT-ADRD topics	Content and skills (all sessions include home-practice of skills)
1	Being in the Here and Now	Deep breathing, mindfulness, and 24-hour block
2	Complex and Conflicting Experiences	Dialectics, mindfulness meditation, and open stress communication
3	Current and Future Challenges	Distress spiral, hands as worries, and acceptance and change
4	Planning and Coping Ahead	Cope ahead, cultivating routines, and coping as a team
5	Difficult Topics	Clarifying goals, effective communication, and relationship repair
6	Relationship Changes	Navigating support and defining roles
7	The Uncertain Future	Goal setting and coping plan
8	Accepting Changes and Making Meaning	Reflection for meaning making

### Assessments

**Figure 2:**
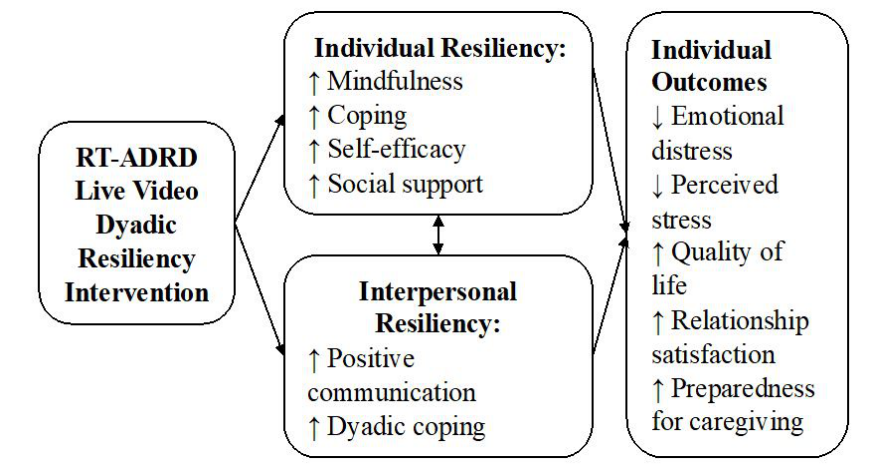
RT-ADRD Conceptual Model.

After consent, both dyad members will independently complete a pretest survey via a secure REDCap link (~30 minutes each) sent by a research coordinator. If needed, participants will also have the option of completing surveys with the assistance of a trained research coordinator via the videoconferencing “Share Screen” tool. The survey will include validated measures to gather information on hypothesized mechanisms of intervention, intervention targets, and treatment moderators, consistent with the overall conceptual model (Figure 2). The primary intervention targets (Table 2, secondary outcomes) are individuals’ emotional distress, relationship quality, and quality of life, which were selected based on their interdependence within dyads and the link of emotional distress to long-term mental and physical health outcomes and relationship functioning [9,14,17,18]. The hypothesized intervention mechanisms (Table 2; described further below) are transdiagnostic resiliency skills that demonstrated links to the same intervention targets in studies of prior dyadic interventions [27]. Following completion of the treatment sessions, dyads will complete a similar posttest survey in REDCap. Responses will be used to compare scores to population norms, examine interrelations among constructs, and inform procedures and the tailoring of RT-ADRD. The primary outcomes for this trial are feasibility and acceptability. In addition, we will use the 6-item Credibility and Expectancy Questionnaire [43] to assess treatment credibility at baseline. We will also use the 3-item Client Satisfaction Questionnaire [44] to assess satisfaction with RT-ADRD at posttest. We will gather comprehensive qualitative feedback on these benchmarks and report detailed information on study screening, enrollment, and intervention deliveries via health care system–supported videoconferencing or in person, to refine feasibility and acceptability benchmarks prior to further testing. A description of study measures is provided below (Table 2).

**Table 2:** Assessment measures.

Construct			Description		Base		Post
**Primary outcomes**							
	Feasibility	Procedures: suitability and practicality of RT-ADRD^a^ and procedures; qualitative feedback from exit interviews Screening: proportion of dyads who screen eligible of those who undergo screening Recruitment and Consent: proportion of dyads who are approached and agree to participate in the intervention Engagement: proportion of dyads who are enrolled and complete 4 out of 6 sessions Assessment: proportion of individuals who are enrolled and complete baseline and post-test assessments		**X**		**X** **X** **X** **X**	
	Acceptability	Credibility and Expectancy Questionnaire (6 items)—assess how credible dyads perceive RT-ADRD Client Satisfaction Questionnaire (3 items)—a measure of satisfaction with RT-ADRD Qualitative feedback from exit interviews		***X***		***X*** ***X***	
**Covariates**							
	Demographics	Review of medical records and participant self-report: age, sex and gender, race and ethnicity, education, and income					
	Clinical Characteristics	Review of medical records and participant self-report: type of ADRDb diagnosis, time since diagnosis, and cognitive tests		**X**			
**Secondary Outcomes**							
	Emotional Distress	Hospital Depression and Anxiety Scale (14 items)—a measure of emotional distress (anxiety and depression subscales) Geriatric Depression Scale (15 items)—a measure of depression symptoms		**X** **X**		**X** **X**	
	Perceived Stress	Perceived Stress Scale (4 items)—a measure of perceived stress		**X**		**X**	
	Quality of Life	WHO Quality of Life-Short Form (26 items)—a measure of quality of life		**X**		**X**	
	Relationship Satisfaction	Couples Satisfaction Inventory (16 items)—a measure of couple relationship satisfaction		**X**		**X**	
	Preparedness for Caregiving (Care partners only)	Preparedness for Caregiving Scale (8 items)- readiness for caregiving in relation to spouse		**X**		**X**	
**Intervention Mechanisms**							
	Mindfulness	Cognitive and Affective Mindfulness-Scale (12 items)—a measure of mindfulness skills		**X**		**X**	
	Coping	Brief COPE (28 items)—a measure of adaptive and maladaptive coping strategies		**X**		**X**	
	Self-efficacy	General Self-Efficacy scale-6 (6 items)—a measure of self-efficacy		**X**		**X**	
	Social Support	Social Support Questionnaire, Short Form—Revised (12 items)—a measure of satisfaction with social support		**X**		**X**	
	Interpersonal Communication and Coping	Dyadic Relationship Scale (10 items—patients, 11 items—caregivers)—a measure of positive and negative dyadic interactions Dyadic Coping Inventory (9 items)—a measure of dyadic stress management		**X** **X**		**X** **X**	

### Covariates

We will collect demographic information on participants’ gender, age, race, ethnicity, marital status, relationship length, education level (number of years in school), employment (status, occupation, and income), and mental health history. We will also collect ADRD clinical characteristics via electronic medical records, including the type of diagnosis, age at diagnosis, and date of diagnosis.

### Secondary Outcomes

#### Overview

We will use the 14-item Hospital Anxiety and Depression Scale (HADS) [40] total score to assess emotional distress once at baseline, and again at the completion of the program. We will examine the 7-item depression and anxiety subscales of the HADS separately (HADS-D and HADS-A, respectively). Scores range from 0 to 21, where a score >7 indicates significant depression or anxiety. We will also use the 15-item Geriatric Depression Scale–Short Form [41], to assess depression symptoms over the past week. Scores range from 0 to 15, with scores ≥5 suggesting the presence of clinically elevated depression symptoms. We will assess perceived stress over the past month using the Perceived Stress Scale [45]. Scores range from 0 to 16, with higher scores indicating greater perceived stress. To assess perceived quality of life, we will use the 26-item brief World Health Organization Quality of Life assessment [46], which captures quality of life across 4 subscales (physical health, psychological, social relationships, and environment). The first 2 items provide a global assessment of quality of life, and the subscale scores are calculated by summing items and transforming scores to a 0 to 100-point interval, with higher scores indicating a greater perceived quality of life. To assess relationship satisfaction, we will use the 16-item Couple Satisfaction Index [47]. Total scores range from 0 to 81, with higher scores indicating greater relationship satisfaction. Finally, for spousal care partners we will use the 8-item Preparedness for Caregiving Scale (9) to evaluate readiness for caregiving in relation to their spouse. Total scores range from 0 to 32, with higher scores indicating greater preparedness for caregiving.

#### Treatment Mechanisms

We selected treatment mechanisms assessing individual and dyadic coping and relationship functioning based on our hypothesized mechanisms underlying changes in proposed outcomes (ie, emotional distress, quality of life). We will use the 12-item Cognitive and Affective Mindfulness Scale, Revised [48], to assess mindfulness. Total scores range from 12 to 48, with higher scores indicating greater engagement in mindfulness practices. We will use the 28-item Brief Coping Orientation to Problems Experienced (Brief COPE) [49], to measure ways of individually coping with stressful events. The Brief COPE includes three subscales that assess coping styles: (1) problem-focused (8 items), (2) emotional-focused (12 items), and (3) avoidant coping (8 items). Scores for each subscale are calculated by taking the mean of all items, with higher scores indicating greater use of that coping style. We will assess perceived self-efficacy with the 10-item General Self-Efficacy Scale [50]. Total scores range from 10 to 40, with higher scores indicating greater perceived self-efficacy. We will use the 12-item brief Social Support Questionnaire (SSQR) [51] to assess social support availability and satisfaction. The SSQR consists of 2 subscales: support network availability (SSQR-N) and overall satisfaction (SSQR-S). Each item assesses support availability and satisfaction in separate parts; participants will be asked to indicate (1) how many people they can count on for various types of support and (2) how satisfied they are with the support. The SSQR-N subscale composite is calculated by taking the mean of all items. For the SSQR-S subscale, total scores are calculated by adding subscale items and range from 6 to 36, with higher scores indicating greater social support satisfaction.

To assess the perceptions of dyadic stress management, we will use the items from the Dyadic Coping Inventory [52,53] subscales: common dyadic coping and negative dyadic coping. The common dyadic coping subscale includes 5 items that assess dyads’ ability to cope with problems together and search for solutions. Scores range from 5 to 25, with higher scores indicating greater perceptions of the dyad’s use of common coping. Negative dyadic coping will also be assessed using the Dyadic Coping Inventory (4 items). Scores range from 4 to 20, with higher scores indicating greater perceptions of the dyads using negative dyadic coping. In addition, we will assess dyadic relationship strain in the context of caregiving using the dyadic strain subscale of the Dyadic Relationship Scale (DRS) [54], which has a 10-item-Patient version and an 11-item Caregiver version. Subscale scores range from 1 to 10 and 1 to 11 for the DRS-Patient and DRS-Caregiver, respectively, with higher scores indicating greater relationship strain.

#### Exit Interviews

At the conclusion of the intervention sessions, dyads will participate together in a single 60-minute exit interview over health care system–supported videoconferencing to gather information on session content and procedures to refine intervention methods prior to the feasibility trial. Topics will include: what was most and least helpful about the intervention, topics or skills that were not included that might be helpful, whether additional sessions would be preferred, perceptions of skills, comfort with participating in sessions together, and barriers and facilitators of engagement in RT-ADRD procedures and the intervention. A trained research assistant will complete the interviews using an interview guide (see Supplemental Materials). As part of the interview, the research assistant will also capture observations within session domains, important quotes, and other important notes using a rapid data analysis template.

### Data Analysis

Feasibility-related measures will be assessed and compared to our prior dyadic intervention studies [27] using proportions at the individual-level on feasibility markers outlined in Table 2, as well as qualitative feedback from exit interviews. Acceptability will be evaluated with the percentage of individuals with scores above the midpoint on self-report questionnaires (Table 2), as well as feedback from exit interviews. Given our primary goal of establishing feasibility and acceptability, this open pilot was not powered to statistically evaluate signal of improvement in quantitative outcomes. However, we will examine preliminary effectiveness of the secondary outcomes using estimated treatment effects for each measure. We will also assess the relationship among treatment mechanisms and secondary outcomes using bivariate correlations, focusing on whether the direction of the association is consistent with our conceptual model. Qualitative data obtained from exit interview transcripts will be analyzed using a rapid data analysis approach to thematic analysis [55]. We will use overarching themes surrounding feasibility markers and participant suggestions to further refine the intervention.

## Results

This study was supported by a grant from the National Institute on Aging (1K23AG075188-02) to SB. It was approved by the institutional review board of the Mount Sinai Hospital in April 2024. Recruitment began in April 2024 and will continue through August 2024. Data collection and analysis is anticipated to be complete by August 2024.

## Discussion

### Anticipated Findings

ADRDs are conditions that are increasingly common among the aging population and have considerable negative impacts on individuals living with ADRD and their spousal care partners [5]. Currently, these dyads have few resources to assist them in navigating early challenges together and adjusting positively to the diagnosis and symptoms [24,25]. Dyads describe an interest in participating in interventions together early after ADRD diagnosis to assist in navigating support and adjusting to symptoms [8]; however, no such interventions exist. Adapting existing interventions has the potential to be effective and efficient in addressing dyads’ challenges to promote dyadic adjustment [27,28], and aid in preventing chronic individual and interpersonal stress. Thus, the RT-ADRD program is a timely potential solution to unmet needs reported by dyads at the time of ADRD diagnosis. The present study aims to refine RT-ADRD, a novel dyadic resiliency intervention for dyads at risk of chronic emotional distress early after ADRD diagnoses that includes individual and interpersonal resiliency training for both dyad members simultaneously to promote adjustment to ADRD.

The larger plan to develop and revise RT-ADRD is guided by the NIH Stage Model for behavioral intervention development [56]. This protocol provides the framework of our open pilot trial of RT-ADRD, stage 1B of the development of RT-ADRD. The intervention is novel and flexible, providing live video delivery of treatment sessions to maximize feasibility and adherence, while also providing the option of in-person sessions upon request. We will use the open pilot results to refine the program and inform the subsequent stage—a larger feasibility trial of RT-ADRD relative to an educational control condition (target N=60 dyads).

### Limitations

Currently, we are only conducting the intervention in English and with couple dyads. This results in important limitations for non–English speakers and for other caregiving dyads commonly facing ADRDs (eg, parent-child dyads). We plan to address these limitations with parallel studies and in subsequent trials by creating a version of the program in Spanish using established guidelines for linguistic adaptation. We also plan to conduct additional qualitative studies to identify adaptations needed to address a range of caregiving dyads.

### Conclusions

The results from this open pilot trial will be used to inform the development of Resilient Together for Dementia, a dyadic resiliency program for those living with ADRDs and their romantic or spousal care partners. Our study will provide quantitative and qualitative data that we will use to refine the current iteration of the program. We will then test the refined intervention in a larger feasibility trial relative to an educational control condition.

## Abbreviations

ADRD: Alzheimer disease and related dementia
Brief COPE: Brief Coping Orientation to Problems Experienced
DRS: Dyadic Relationship Scale
HADS: Hospital Anxiety and Depression Scale
HIPAA: Health Insurance Portability and Accountability Act
MoCA: Montreal Cognitive Assessment
NIH: National Institutes of Health
REDCap: Research Electronic Data Capture
RT-ADRD: Resilient Together for Dementia
SSQR: Social Support Questionnaire
SSQR-N: Social Support Questionnaire-network availability
SSQR-S: Social Support Questionnaire-overall satisfaction

## Data Availability

Data collection and analysis instruments are available in the Multimedia Appendices. The datasets generated during and/or analyzed during the current study are available from the corresponding author on reasonable request.
